# Deep Cervical Muscles and Functionality in Patients with Chronic Tension-Type Headache: An Observational Study

**DOI:** 10.3390/medicina58070917

**Published:** 2022-07-10

**Authors:** Guillermo García-Pérez-de-Sevilla, Ángel Gónzalez-de-la-Flor, Daniel Martín-Vera, Diego Domínguez-Balmaseda, José Ángel del-Blanco-Muñiz

**Affiliations:** Department of Physiotherapy, Faculty of Sports Sciences, Universidad Europea de Madrid, 28670 Madrid, Spain; angel.gonzalez@universidadeuropea.es (Á.G.-d.-l.-F.); daniel.martin2@universidadeuropea.es (D.M.-V.); diego.dominguez@universidadeuropea.es (D.D.-B.); joseangel.delblanco@universidadeuropea.es (J.Á.d.-B.-M.)

**Keywords:** tension-type headache, deep cervical muscles, muscle thickness, pain vigilance

## Abstract

*Background and objectives:* Chronic tension-type headache (TTH) is the type of headache with the highest prevalence. The involvement of musculoskeletal structures in TTH is supported by evidence in the scientific literature. Among these, deep cervical muscle strength appears to be related to the function of the cervical spine and the clinical characteristics of TTH. This study aimed to correlate anatomical, functional, and psychological variables in patients with TTH. *Materials and methods:* An observational descriptive study was carried out with 22 participants diagnosed with TTH for at least six months. The characteristics of headaches, including ultrasound-based deep neck flexor and extensor muscle thickness, range of motion (ROM), and pressure pain threshold (PPT), were recorded. We also conducted the Pain Vigilance and Awareness Questionnaire (PVAQ) and the Craniocervical Flexion Test (CCFT). *Results:* Moderate–large negative correlations were found between the PVAQ and the muscle thickness of right deep flexors contracted (r = −0.52; *p* = 0.01), left multifidus contracted (r = −0.44; *p* = 0.04), right multifidus at rest (r = −0.48; *p* = 0.02), and right multifidus contracted (r = −0.45; *p* = 0.04). Moderate–large positive correlations were found between the CCFT score and the left cervical rotation ROM (r = 0.53; *p* = 0.01), right cervical rotation ROM (r = 0.48; *p* = 0.03), muscle thickness of left multifidus contracted (r = 0.50; *p* = 0.02), and muscle thickness of right multifidus at rest (r = 0.51; *p* = 0.02). The muscle thickness of the contracted right deep cervical flexors showed a moderate negative correlation with headache intensity (r = −0.464; *p* = 0.03). No correlations were found between PPT and the rest of the variables analyzed. *Conclusions:* In patients with TTH, a higher thickness of deep cervical muscles was associated with higher ROM and higher scores in the CCFT. In turn, the thickness of deep cervical muscles showed negative correlations with pain hypervigilance and headache intensity. These results contribute to a better understanding of the physical and psychosocial factors contributing to the development of TTH, which is useful for implementing appropriate prevention and treatment measures.

## 1. Introduction

Headaches are a public health problem and one of the most common symptoms worldwide [1]. According to the Global Burden of Disease, the global prevalence of active headache disorders is 52%. Among all types of headaches, TTH is the most prevalent, representing 26% of all diagnosed headaches [2].

Despite efforts made by the International Headache Society (IHS) to clarify the diagnostic criteria for each type of headache, which is established in the latest edition of the International Classification of Headaches [3], nonspecific criteria are followed to accurately diagnose TTH, which makes its diagnosis more difficult.

Clinically, TTH pain is described as bilateral, dull, and oppressive, and can range from mild to moderate intensity [3]. Recent studies have shown that some associated symptoms such as osmophobia [4] or comorbid disorders, such as depression [5], are quite similar to those described in migraines. Regarding the pathophysiology of TTH, its origin is considered to be multifactorial, with four factors involved: (i) genetic factors: an increased risk of TTH was observed in first-degree relatives [6]; (ii) environmental factors: stress, anxiety, and depression are some of the most frequent triggers of TTH [7]; (iii) peripheral mechanisms: patients with TTH show increased sensitivity to pericranial muscle pressure due to activation of peripheral nociceptors, with higher presence of myofascial trigger points [8]; (iv) central mechanisms: patients with chronic TTH present a decrease in pain threshold to nociceptive stimulus from pericranial myofascial structures, due to sensitization of the dorsal horn neurons and the trigeminocervical nucleus affecting cranial and extracranial levels [9]. In addition, patients with TTH have decreased nociceptive inhibitory mechanisms and overactivated pericranial muscles [10].

The involvement of musculoskeletal disorders in patients with TTH is sufficiently demonstrated in the scientific literature. Common exploratory findings in these patients are the presence of myofascial trigger points in the cranial and pericranial muscles [8], presence of neck pain [11], and weakness of the deep cervical muscles [12]. A decrease in deep cervical muscle thickness was also demonstrated in TTH patients [13], which may be related to a greater weakness in deep neck flexor muscles, tested by CCFT [14]. The correlation mentioned above was demonstrated in one study involving cervical degenerative disorders [15], and furthermore in distal regions such as the quadriceps in healthy participants [16]. Concerning treatment, supervised physical activity interventions might have positive effects on pain intensity and headache frequency [17].

In pathologies where pain becomes chronic, psychological affectations seem to be linked. In order to examine this association, one of the most commonly used scales is the Pain Vigilance and Awareness Questionnaire (PVAQ) [18]. A recent study showed that higher pain levels in patients with bruxism were related to higher anxiety and stress results [19]. Moreover, high anxiety levels were also associated with a higher chance of reporting headaches [20].

In addition, some authors reported correlations between functional aspects, such as muscle strength, and psychological affectation derived from pain. Thus, a study performed in patients with chronic knee osteoarthritis concluded that a muscle strength program had a positive effect on psychological aspects such as anxiety [21]. These data have direct implications for the management of patients with chronic TTH, setting the scope beyond musculoskeletal structures, which enables the addition of psychological aspects and pain education [22].

As such, the objective of this study was to analyze the relationship between anatomical and functional variables, pain characteristics, and its psychological impact in patients with chronic TTH.

## 2. Methodology

An observational study following the Strengthening the Reporting of Observational Studies in Epidemiology (STROBE) Initiative Statement [23] was conducted with a sample of patients diagnosed with TTH for more than six months.

The study protocol adhered to the principles of the 1964 Declaration of Helsinki and its subsequent clarifications, and was approved by the Research Ethics Committee of the Rey Juan Carlos University of Madrid (reference number: 1802202105721). Additionally, informed consent was obtained from all subjects involved in the study.

### 2.1. Participants

Recruitment of participants (n = 22) was carried out among students and workers at a university. Participants were included in the study who fulfilled the following criteria: (1) adults aged 18–65 years; (2) having TTH for more than six months (diagnosed with chronic TTH by their neurologist, following the criteria of the International Headache Society’s classification of headaches, in its third edition). The exclusion criteria were: (1) the presence of pathologies that prevent the performance of physical activity; (2) pregnancy.

### 2.2. Variables

Anthropometric variables were age in years, height in centimeters (cm), and weight in kilograms (kg). Height was measured with a measuring rod (Ano Sayol SL, Barcelona, Spain), and weight with a mechanical scale (Asimed T2, Barcelona, Spain). Body mass index (BMI) was calculated as weight (kg)/height (m^2^) following Shephard’s protocol [24].

#### 2.2.1. Characteristics of Headache Episodes

The protocol of Gago-Veiga et al. was followed to measure the duration of the headaches (expressed in hours), the intensity of the tension headache (on the numeric pain rating scale from 0 to 10), and the frequency (in days) of the episodes of the subject [25].

#### 2.2.2. Muscle Thickness

**Figure 1:**
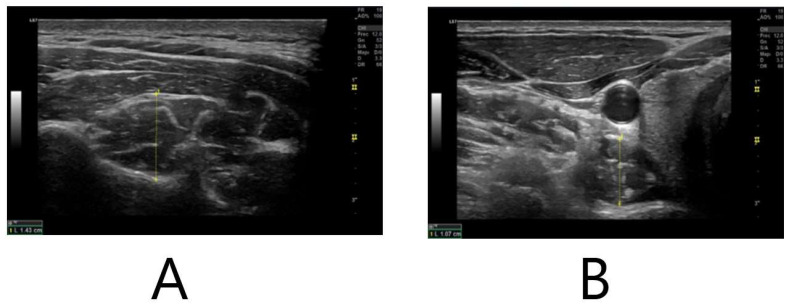
Ultrasound assessment of deep cervical muscles. (**A**) Ultrasound image of the multifidus in transverse view at C5. The caliper is placed 90⁰ to the lamina of C5 where the rater considered the muscle to be at its thickest and up to the echogenic line of the hyperechoic fascia between the semispinalis cervicis and semispinalis capitis. (**B**) Ultrasound image of the longus colli at C6 transverse view. The caliper is placed on the midpoint of the ventral surface of the C6 vertebral body and the interface between the Lcol and the pre-fascial tissue surrounding the carotid artery.

Measurements of the thickness of the neck stabilizing muscles flexor longus colli and multifidus at the cervical C5–C6 level (Figure 1) were recorded by ultrasonography (GE Healthcare, Chicago, IL, USA) at rest and counter-resistance following the methodology of Øverås et al. [26]. The participants were asked to remain relaxed when taking the measurements. In order to ensure this, a physiotherapist from the research team checked with palpation that the subjects’ muscles did not show signs of contraction.

In the counter resistance measurement, the participants were asked to perform a “double chin”, bringing their chin to their sternal notch. For this protocol, the subjects previously performed a familiarization that the physiotherapist demonstrated.

#### 2.2.3. Range of Motion

The cervical range of motion (ROM) was analyzed with a CROM goniometer Baseline (Fabrication Enterprises INC., New York, NY, USA). The ranges evaluated were ROM Left Rotation, ROM Right Rotation, ROM Flexion, ROM Extension, ROM Tilt Left, and ROM Tilt Right, all measured in degrees. The procedure was followed as in the study by Wolan-Nieroda et al., in which the subjects were kept in a seated position to perform the measurements, and data were collected of the mentioned ranges. Measurements made with the CROM goniometer showed inter- and intra-rater agreement on cervical ROM assessments. Therefore, the CROM goniometer is a reliable instrument for use in daily clinical practice [27]. The test-retest reliability of the measurements made with the CROM was verified, with ICC values for all cervical measurements ranging between 0.89 (CI: 0.73–0.96) for flexion and 0.98 (CI: 0.95–0.99) for extension, showing good validity [28].

#### 2.2.4. Pressure Pain Threshold (PPT)

The protocol and methodology of the Fleckenstein et al. study was followed. A FORCE DIAL FDK/FDN 100 algometer (Wagner Instruments, Greenwich, CT, USA) was taken as the measurement tool. PPT is known to be the most validated mechanical threshold at present [29].

**Figure 2:**
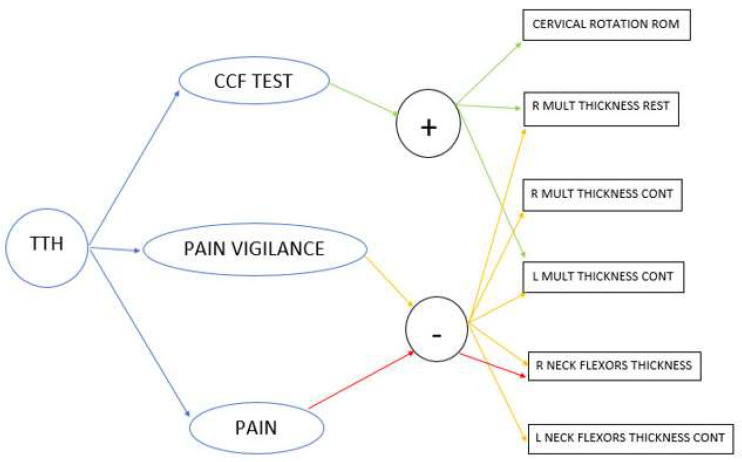
Correlations between the variables. Positive (+) and negative (−) correlations are shown from TTH (Tension-type headache) with craniocervical flexion (CCF) test, Pain vigilance and levels of headache. Abbreviations: R (right), L (left), Mult (Multifidus), Cont (in contraction).

Participants were assessed on a stretcher by a physiotherapist (Figure 2). PPT measurements were performed bilaterally in the following regions: upper trapezius, masseter, and temporalis [26]. Each muscle was analyzed with the participants lying down in the supine position. The participants were informed that the investigators would perform mechanical pressure on different regions of the body to analyze the PPT, so as to quantify it through an algometer. The PPT measurements using algometry showed good-to-excellent interrater reliability (ICC: 0.64–0.92) and test-retest reliability (ICC: 0.72–0.95) [30].

#### 2.2.5. Pain Vigilance and Awareness Questionnaire (PVAQ)

The PVAQ is an internationally accepted tool to evaluate this characteristic; in 2013 it was validated and translated into Spanish by Esteve et al. [31]. It consists of a 16-item self-reported questionnaire that measures the frequency of self-controlled and self-reported attention habits, with a focus on pain and changes in pain during the last 2 weeks. PVAQ was carried out following the study by Martinez et al., in which the subjects would respond to each item, using the following numerical scale: 0 = never, 1 = sometimes, 2 = half the time, 3 = frequently, 4 = almost always, and 5 = always [18]. This instrument was psychometrically evaluated and showed good internal consistency (Cronbach’s alpha = 0.86), and adequate test-retest reliability (r = 0.80), which supported the construct validity of the PVAQ [32].

#### 2.2.6. Craniocervical Flexion Test (CCFT)

The CCFT was carried out to analyze the functionality of the cervical musculature; for this procedure, we used a Stabilizer Pressure biofeedback tool (Chattanooga Group, Hixon, EE.UU.), following the study of Thongprasert and Kanlayanaphotporn [33]. The participants were placed supine with both knees bent and the cervical spine in a neutral position. The forehead and chin were horizontally aligned with the surface of the socket. The Stabilizer was placed behind the neck, in the suboccipital region, before being inflated to the initial pressure of 20 mmHg. Participants were instructed to perform a slow and controlled craniocervical flexion in a nodding action, progressively increasing the pressure in 2 mmHg increments from 20 to 30 mmHg, and maintaining it in each increment for 10 s to 30 s. Rest was allowed between successful raises. During testing, the contraction of the superficial neck flexor muscles was palpated by an assessor whose activity was kept to a minimum. Two data were recorded during the CCFT, i.e., the activation score (AS) and the performance index (PI). The AS was defined as the highest pressure-level change the participants could achieve and maintain steadily for 10 s. The PI, which reflects the isometric endurance of the deep cervical flexor muscles, was calculated by multiplying the number of times the participants could replicate the test at the AS. The highest score of the PI was set at 100 (10 repetitions at 10 mmHg AS). Based on the previous studies in people with and without neck pain, these data were converted into a rating in which an abnormal response for the AS and the PI were ≤ 4 mmHg and ≤ 20 scores, respectively [34]. The ICC value for inter-rater reliability was 0.89 with a 95% confidence interval (0.70–0.94). The ICC value for intrarater reliability was 0.87 with a 95% confidence interval (0.77–0.93), so the Stabilizer showed good-to-excellent inter- and intrarater reliability [35].

All the measurements were performed by a research team composed of six physiotherapists, two of them specialized in TTH. Each measurement was supervised by at least two physiotherapists to avoid any bias and poor execution of the techniques.

### 2.3. Statistical Analysis

First, the Shapiro–Wilk test was performed to assess the normality of distribution for all the continuous variables (headaches characteristics, ROM, muscle thickness, PPT, CCFT, and PVAQ) [36]. Second, the descriptive analysis was carried out for all the participants using mean ± standard deviation (SD). Third, to analyze the relationship between continuous variables, the Spearman correlation test and the Pearson correlation test were performed for the nonparametric and the parametric variables, respectively. The magnitudes of correlation between continuous variables were qualitatively interpreted using the following criteria: trivial (r ≤ 0.1), small (r = 0.1–0.3), moderate (r = 0.3–0.5), large (r = 0.5–0.7), very large (r = 0.7–0.9), and almost perfect (r ≥ 0.9) [37]. Otherwise, correlation was interpreted as the observed magnitude. Finally, a multiple linear regression was performed among variables that already showed significant correlations. The statistical significance was set at an alpha level of <0.05. All analyses were conducted using IBM SPSS for Windows (version 25, IBM Corporation, Armonk, NY, USA).

## 3. Results

### 3.1. Sociodemographic Data of the Sample

A total of N = 22 participants, mainly women (82%), with chronic TTH were analyzed. The mean age was 39.72 ± 13.91 years, and the mean BMI was 25.01 ± 4.24 kg/m^2^.

### 3.2. Headache’s Characteristics

The mean intensity of the headaches was 7.11 ± 1.33 on a numeric pain rating scale from 0 to 10. These headaches had a mean duration of 18.91 ± 20.23 h per day, and a mean frequency of 13.00 ± 10.78 days per month.

### 3.3. Muscle Thickness

Ultrasound imaging measurements of the thickness of left multifidus, right multifidus, right deep flexors, and left deep flexors are described in Table 1. All measurements were performed during the contraction and relaxation phase.

### 3.4. Range of Motion

The ROMs of cervical flexion, extension, left rotation, right rotation, left inclination, and right inclination are described in Table 1.

### 3.5. Pain Pressure Threshold

The PPTs of the right temporalis, left temporalis, right trapezius, left trapezius, right masseter, left masseter, right median nerve, and left median nerve are described in Table 1.

PVAQ and CCFT scores are described in Table 1.

### 3.6. Correlations between the Continuous Variables

Moderate–large negative correlations were found between PVAQ and the following variables: right deep flexors contracted muscle thickness (r = −0.52; *p* = 0.01), left multifidus contracted muscle thickness (r = −0.44; *p* = 0.04), right multifidus muscle thickness (r = −0.48; *p* = 0.02), and right multifidus contracted muscle thickness (r = −0.45; *p* = 0.04) (Figure 2).

In turn, moderate–large positive correlations were found between the CCFT and the following variables: left cervical rotation ROM (r = 0.53; *p* = 0.01), right cervical rotation ROM (r = 0.48; *p* = 0.03), left multifidus contracted muscle thickness (r = 0.50; *p* = 0.02), and right multifidus muscle thickness (r = 0.51; *p* = 0.02) (Figure 2). In the multiple linear regression, the CCFT score showed a significant relationship with the right multifidus muscle thickness (R^2^ = 0.225; *p* = 0.02) and the left cervical rotation ROM (R^2^ = 0.180; *p* = 0.04).

Finally, right deep flexors’ contracted muscle thickness showed a moderate negative correlation with the headache intensity (r = −0.464; *p* = 0.03). No correlations were found between PPT and the rest of the variables analyzed (Figure 2).

## 4. Discussion

The aim of the present study was to analyze the associations between headache characteristics, physical impairments, and psychological aspects, such as hypervigilance, in patients with TTH. This novel research provides useful information for understanding the physical and psychosocial factors contributing to the development of TTH, which could be useful for implementing appropriate prevention or treatment measures.

In the present study, there were large positive correlations between the CCFT performance and the thickness of right and left multifidus in contraction. Other authors have reported similar results. Ishida et al. [38], analyzed the activation of the sternocleidomastoid muscle during the test performance; they concluded that there is a negative relationship between the thickness of the deep cervical musculature and its activation in healthy patients at 26 mmHg (r = −0.622; *p* = 0.023) and 28 mmHg (r = −0.653; *p* = 0.015) pressure values. Similar results were obtained in the study conducted by Jull and Falla [39], who analyzed the electromyographical activity of the deep and superficial flexor muscles in patients with neck pain during the CCFT, and found a moderate negative correlation between these variables (r = −0.34; *p* < 0.01).

Likewise, in this study, there was a negative correlation between deep cervical muscles thickness and pain intensity. The role of cervical musculature disorders in patients with TTH seems crucial [40]. Previous studies showed how deep cervical muscle weakness is associated with greater cervical pain, and how programs that strengthen these muscles improve clinical variables in patients with TTH [41].

In addition, another finding was a positive correlation of cervical spine rotational ROM and the CCFT score. Previous studies in people with chronic neck pain, cervico-craniofacial pain, and temporomandibular disorders have reported similar values for neck axial rotation ROM [42,43]. These findings imply that achieving an adequate axial rotation ROM may improve the function of the deep cervical muscles.

The participants of this study had a total axial rotation (right + left) ROM of 127.77°, which is less than other results reported with TTH patients (mean = 146.15°) [44,45]. In line with our findings, Liang et al. [11] found that participants with TTH had less cervical ROM compared with healthy subjects. However, the lack of a control group in the present study necessitates caution in the interpretation of these results [46,47].

The lack of association between sagittal (flexion + extension) plane ROM and other variables can be explained due to absence of physical impairment compared with normal values in asymptomatic participants [44,45]. No association was found between the total frontal (right + left) plane ROM and other variables, but the participants showed a reduced frontal plane ROM (mean = 70.2°), according to the results of several studies in TTH patients [44,45]. Our results showed less total frontal plane ROM compared with other studies [48,49].

Furthermore, the lack of association in our study between cervical ROM, headache features, or deep cervical muscle thickness with pressure sensitivity of cranial and pericranial points supports the possibility that cervical impairments can sometimes be unrelated to headache and, in some patients, be incidental features or co-existing cervical disorders [50]. One systematic review revealed that no significant associations were found between PPT values in the cranio-cervical sites, and headache characteristics such as frequency, duration, or intensity. The increased sensitivity of cranio-cervical sites supports the neurophysiological model of sensitization in migraine and chronic TTH [51]. In addition, no association between PPT values and other variables could be explained due to the methods used in the study (mechanical device rather than electronic) [52].

Concerning the characteristics of the sample, most participants in the present study were women (82%). Female sex is associated with an increased risk of reporting headache, according to recent health surveys [20].

Finally, this is the first study to investigate the relationship between hypervigilance and the thickness of the deep neck muscles. Previous studies have linked hypervigilance to people with fibromyalgia and knee osteoarthritis [53,54,55]. Hypervigilance leads to an increased ability to detect potentially harmful stimuli. Regarding pain, hypervigilance results in increased attention to pain and pain-related information. Although it represents an adaptation in the acute stage of pain, hypervigilance in chronic or recurrent pain states can lower pain detection thresholds and increase pain-related interference [18,55]. This theory may explain the relationship between hypervigilance and the reduction in the deep neck muscles’ thickness in people with TTH.

However, no correlations between anatomical and functional variables (thickness or ROM) with pressure sensitivity of cranial and pericranial points were found, unlike other previous investigations [50].

Several limitations associated with this study should be acknowledged. First, an observational study was used to explore the associations, but the relationships cannot be inferred from the results. Second, a limitation of the study is the lack of a control group to compare the results with those of TTH patients. Third, only the C5 cervical segment was measured to determine the cervical multifidus thickness. Finally, it would be interesting for future studies to investigate the associations between another psychosocial element and health-related quality of life in TTH patients.

## 5. Conclusions

In patients with TTH, the CCFT performance showed large positive correlations with the thickness of deep cervical muscles, which in turn had large negative correlations with pain vigilance and headache intensity. In addition, there was also a direct correlation between the CCFT performance and the cervical rotation ROM, but with the lateroflexion and flexion-extension ROM. These results contribute to an understanding of the physical and psychosocial factors contributing to the development of TTH, which is useful for implementing appropriate prevention or treatment measures.

## Figures and Tables

**Table 1:** Descriptive analysis of the variables analyzed.

Variables		Mean ± SD
**Range of Motion (degrees)**	Left rotation	62.36 ± 10.63
	Right rotation	65.41 ± 6.91
	Flexion	61.91 ± 15.28
	Extension	60.59 ± 11.25
	Left inclination	35.45 ± 6.92
	Right inclination	34.73 ± 6.98
**Muscle thickness** **(cm)**	Right multifidus	1.07 ± 0.22
	Right multifidus contracted	1.23 ± 0.17
	Left multifidus	1.13 ± 0.19
	Left multifidus contracted	1.25 ± 0.20
	Right deep flexors	1.01 ± 0.18
	Right deep flexors contracted	1.21 ± 0.19
	Left deep flexors	1.03 ± 0.16
	Left deep flexors contracted	1.18 ± 0.19
**Pain Pressure Threshold (lb/cm^2^)**	Right temporalis	3.29 ± 1.34
	Left temporalis	2.96 ± 1.19
	Right upper trapezius	2.96 ± 1.19
	Left upper trapezius	3.79 ± 1.67
	Right masseter	2.81 ± 1.12
	Left masseter	2.76 ± 1.42
Cervical flexion test (mmHg)		21.33 ± 1.83
Pain vigilance and awareness questionnaire		27.91 ± 7.27

## Data Availability

Data available upon request due to ethical and privacy restrictions.
